# Polish regulatory system regarding ATMP hospital exemptions

**DOI:** 10.3389/fimmu.2024.1379134

**Published:** 2024-05-13

**Authors:** Jan Pachocki, Frances Verter

**Affiliations:** ^1^Consultant, Warsaw, Poland; ^2^Parent’s Guide to Cord Blood^®^ Foundation, Brookeville, MD, United States

**Keywords:** advanced therapy medicinal products (ATMPs), cell and gene therapy (CGT), hospital exemptions (HE), mesenchymal stromal cells (MSC), regulatory affairs, national licensing provisions, risk -benefit, unproven cell and gene interventions

## Abstract

**Introduction:**

This article explains the current regulatory system in Poland regarding Advanced Therapy Medicinal Products given under Hospital Exemptions (ATMP-HE).

**Methods:**

The relevant sections of Polish legislation are translated into English and their interaction is described.

**Results:**

We analyze the impact of these regulations from the perspective of three stakeholder groups: manufacturers, physicians, and patients. Amendments enacted between 2018 and 2023 have substantially changed Polish implementation of the ATMP-HE pathway. In Poland, most ATMP-HE treatments have been therapies employing Mesenchymal Stromal Cells (MSC).

**Discussion:**

Comparison to other European countries shows that Poland is within the mainstream of EU practices regarding ATMP-HE implementation. One notable issue is that Poland has relatively low per capita spending on healthcare, and ATMP-HE in Poland must be funded from outside the government healthcare system. Conclusions. The original intention of the legislation that created ATMP-HE was to allow access to experimental therapies for patients with unmet needs. It remains to be seen if that mission can be fulfilled amidst conflicting pressures from various stakeholder groups.

## Graphical Abstract

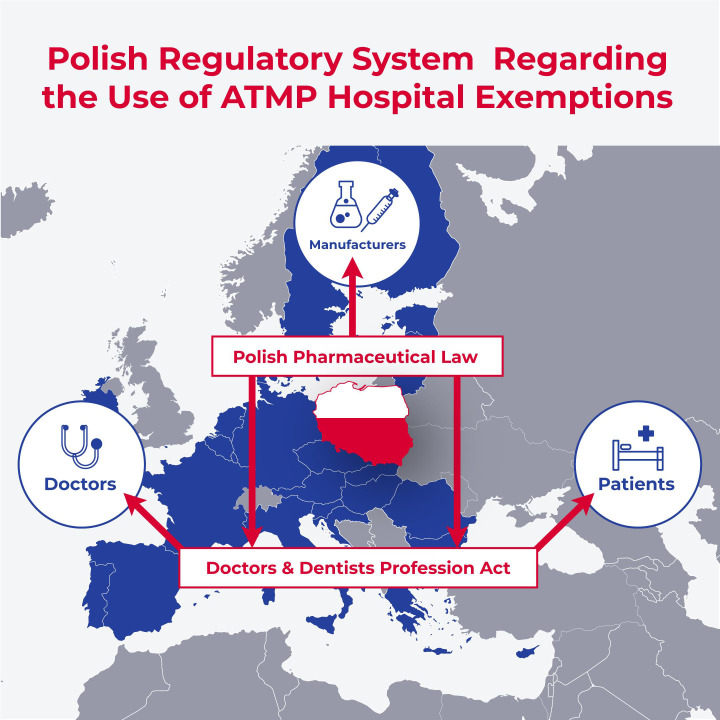
Schematic diagram of the Polish regulatory system for Advanced Therapy Medicinal Products given under Hospital Exemptions (ATMP-HE). The three main stakeholders in the ATMP-HE framework of Poland are manufacturers of ATMP, doctors that prescribe ATMP, and patients that are treated with ATMP. The analysis of ATMP-HE regulations in terms of these three stakeholder groups can be applied to other countries. The two main laws that regulate ATMP-HE in Poland are the Polish Pharmaceutical Law, and the Doctors and Dentists Profession Act. In each country, the interactions between doctors and patients are governed by regulations on healthcare providers, so that any national policies on ATMP-HE must pass through a second layer of regulation before they reach these stakeholders.

## 1 Introduction

The European Medicines Agency (EMA) is an agency of the European Union (EU) which is responsible for the scientific evaluation, supervision, and safety monitoring of medicines in the EU (1). The medical regulations from the EMA provide guidelines for member states, but leave flexibility within those guiding limits for the path traveled by individual nations. For example, only the authorities in individual member states can authorize clinical trials of advanced therapy medicinal products (ATMP), or grant final market approval of ATMP within their borders (1). Hospital exemptions (HE) are another category of medical treatment where the role of the EMA overlaps with, but does not replace, the regulatory role of competent authorities in each EU member state. The European parliament adopted Regulation 1394/2007/EC that amended Directive 2001/83/EC and created a hospital exemption pathway for ATMPs (2). The hospital exemptions created in Regulation 1394/2007/EC were intended to provide expanded access to experimental therapies for patients with unmet needs. While on the one hand the ATMP-HE pathway allows access to experimental products that are not yet approved, on the other hand it creates a safe space for the use of those products by imposing quality controls on the product manufacturing and safety monitoring of the clinical delivery (2).

Since the establishment of the ATMP-HE pathway in 2007, its application has evolved with the development of new therapies. While the language of Regulation 1394/2007/EC constrains the manufacturing conditions, it does not restrict the patients’ indications for use. A traditional justification for HE therapies has been “compassionate use” therapies for patients that have incurable conditions for which there is no therapy with marketing authorization. Subsequently, newer cell and gene therapies like CAR-T have achieved market authorization, and currently the ATMP-HE pathway is also used to allow patients to access these new therapies in circumstances where they do not qualify for the standard treatment protocols (3–5).

One source of creative tension in the application of the ATMP-HE pathway is that each EU member country has developed their own method of interpreting and applying the ATMP-HE regulations. As a result, the landscape of what therapies are covered by ATMP-HEs and how these treatments are offered may differ significantly from one country to another, as previous authors have noted (3–15). Studies of the sociology of science have pointed out that scientists and regulators may feel pressures to modify universal standards to their own national circumstances, either because of a need to adapt to local realities like pre-existing practices, or out of a desire to be competitive with other nations (16–18). Some members of the cell and gene therapy (CGT) community have argued that the ATMP-HE regulations in various countries should be more harmonized (5, 13, 14), whereas others feel that the current system encourages innovation, localization, and cost savings (10, 12, 15).

Another source of tension and outright controversy is that ATMP-HE have become somewhat politicized. The arena of regenerative medicine is plagued by the existence of illegal clinics which advertise direct to consumers that their alleged stem cell products will cure anything that ails a patient (19–21). There has been a rallying cry among alliances of pharmaceutical companies and societies of CGT professionals that there is a need to stamp out “unproven” therapies (21). Those patients that receive ATMP-HE composed of Mesenchymal Stromal Cells (MSC) are often receiving treatments that are very similar to what is offered in illegal clinics, except they are subject to the ATMP-HE requirements on quality control of the manufacturing process and supervision of the clinical safety. Nonetheless, the fact that ATMP-HE products are not “proven” therapies has led some authors to place them in the same bucket as “unproven” commercialization (14). For this reason, the use of ATMP-HE should be evaluated in the context of community standards for the safety and efficacy of MSC therapies (22–24).

## 2 Methods

The purpose this paper is to explain in detail the current requirements and application of the ATMP-HE regulations in Poland. This paper starts by providing English translations of the key sections of Polish law that govern the availability and application of ATMP-HE programs. In addition, we explain how these laws are interpreted and applied in the context of the Polish healthcare system. We make note of several amendments to these laws which have been adopted recently and which are expected to significantly alter ATMP-HE practices in Poland going forward.

This is the first paper on ATMP-HE which analyzes the impact of the regulatory system from the perspective of three main stakeholder groups: manufacturers, physicians, and patients in Poland. This approach can be generalized to other countries. The breakdown into stakeholder groups helps to illustrate that there are multiple national regulations that filter ATMP-HE policies before they are applied. For example, interactions between healthcare providers and patients are always regulated by national authorities. These layers contribute to any variations that are seen between ATMP-HE practices in different countries.

In the discussion section we compare ATMP-HE practices in Poland to several other European countries for which we have accessed the regulations, as well as to previous publications on ATMP-HE variations among EU countries.

## 3 Results

### 3.1 Legal basis of using ATMP-HE under Polish Law

The existence of Hospital Exemptions in the European Union was established by Article 28 of EU Regulation 1394/2007/EC, which introduced modifications to the previous Article 3 Directive 2001/83/EC on medicinal products for human use (2). The new Article 28 implemented special conditions that allow patients to be treated with advanced therapy medicinal products that have not received marketing authorization. Article 28 also outlined the criteria necessary to apply for this exemption. This exemption is called ATMP Hospital Exemption or ATMP-HE.

The exact language of Article 28 of Regulation 1394/2007/EC that creates ATMP-HE pathway states as follows in English: *“Any advanced therapy medicinal product, as defined in Regulation (EC) No 1394/2007/EC, which is prepared on a non-routine basis according to specific quality standards, and used within the same Member State in a hospital under the exclusive professional responsibility of a medical practitioner, in order to comply with an individual medical prescription for a custom-made product for an individual patient. Manufacturing of these products shall be authorized by the competent authority of the Member State. Member States shall ensure that national traceability and pharmacovigilance requirements as well as the specific quality standards referred to in this paragraph are equivalent to those provided for at Community level in respect of advanced therapy medicinal products for which authorization is required pursuant to Regulation (EC) No 726/2004/EC of the European Parliament and of the Council of 31 March 2004 laying down Community procedures for the authorization and supervision of medicinal products for human and veterinary use and establishing a European Medicines Agency”* (2, 25).

After the 2007 EU regulations establishing ATMP-HE, regulations in Poland regarding the use of medicinal products were modified on 18 March 2011. This was done through the Act that established the President of the Office for Registration of Medicinal Products, Medical Devices and Biocidal Products. Amendments were made by adding Article 3 Section 4 Point 7 to Poland’s Pharmaceutical Law (26). The current language of this law and its amendments date from June 2018 (27). Prior to the Amendment of the Pharmaceutical Law in June 2018, there was no obligation for hospitals in Poland to offer ATMP-HE, which was a major legislative defect.

Currently, the Pharmaceutical Law of Poland reads as follows: “*an advanced therapy medicinal product - hospital exception - is an advanced therapy medicinal product within the meaning of Art. 2 sec. 1 lit. and Regulation (EC) No. 1394/2007/EC of the European Parliament and of the Council of 13 November 2007 on advanced therapy medicinal products and amending Directive 2001/83/EC and Regulation (EC) No. 726/2004/EC (Official Journal EU L 324 of 10.12.2007, p. 121, as amended13), hereinafter referred to as ‘Regulation 1394/2007’, which is prepared in the territory of the Republic of Poland on a non-routine basis in accordance with quality standards and applied as part of hospital medical services within the meaning of Art. 2 sec. 1 point 11 of the Act of 15 April 2011 on medical activity (Journal of Laws of 2022, items 633, 655, 974 and 1079) in the territory of the Republic of Poland under the sole responsibility of a physician in order to make an individually prescribed medicinal product for a given patient.”* (27).

**Table 1** presents an analysis that compares the concepts of EU Article 28 of Regulation 1394/2007/EC versus Poland’s Pharmaceutical Law implementing the ATMP-HE provision (2, 25–27). There are differences in the Polish language that appears instead of the three EU quotes “custom-made product for an individual patient”, “used in a hospital”, and “under the exclusive professional responsibility of a medical practitioner” (23–27). We discuss these in turn. First, the Polish Pharmaceutical Law does not include the term “custom-made”, only indicating a similar but broader term “individually prescribed” (26, 27). In Poland the requirement to customize the treatment is implicitly included when the doctor individually prescribes the ATMP-HE product. Second, while some EU member states interpret “used in a hospital” loosely to include clinics, under the current Polish system of providing healthcare services, ATMP-HE may only be administered in hospitals and not in outpatient facilities (28). Third, the EU phrase “medical practitioner” has become “doctor” because in Poland only doctors may prescribe ATMP-HE under the regulations of the Doctor and Dentist Profession Act (29, 30). The responsibilities of doctors prescribing ATMP-HE in Poland are discussed more below.

**Table 1:** Language of ATMP-HE regulations under EU Law versus Polish Law.

EU Article 28 Regulation 1394/2007	Poland Pharmaceutical Law	Comment
*“prepared … and used within the same Member State”*	*“which is prepared in the territory of the Republic of Poland … and applied …. in the territory of the Republic of Poland”*	No significant differences.
*“used in a hospital”*	*“applied as part of hospital medical services within the meaning of Art. 2 sec. 1 point 11 of the Act on Medical Activity”*	The Polish Pharmaceutical Law was amended in June 2018 to make it clear that a hospital is obligated to offer ATMP-HE as part of hospital service (27). According to the Polish Act on Medical Activity, a hospital is a medical facility that provides inpatient care, as opposed to outpatient services (28).
*“under the exclusive professional responsibility of a medical practitioner”*	*“under the sole responsibility of a physician”*	Polish law has narrowed the term ‘medical practitioner’ that appears in EU regulations. Polish regulations limit the administration of ATMP-HE to a doctor, as under Polish regulations only a doctor is competent to administer this medicinal product.
*“prepared on a non-routine basis according to specific quality standards”*	*“prepared on a non-routine basis in accordance with quality standards”*	No significant differences, merely some editorial changes in language. The supervision over the manufacturing of ATMP-HE are discussed in **Table 2**. The fact that EU Regulations do not define the concept of “non-routine basis” should be regarded as a deliberate omission which enables the EU member states to make interpretations in the context of each specific health care system. In Poland, the national regulator also has not issued any official guidelines or restrictions for “non-routine basis”. But the existence of “non-routine” circumstances are indirectly covered when the manufacturer applies to the Polish Minister of Health for permission to deliver ATMP-HE therapy. Each application for ATMP-HE consent is assessed on the basis of the specific situation.
*“in order to comply with an individual medical prescription for a custom-made product for an individual patient”*	*“in order to make an individually prescribed medicinal product for a given patient”*	It is important to note that the official Polish translation of EU Article 28 of Regulation 1394/2007 reads slightly differently from the English translation, because it omits the phrase “custom made.” However, the Polish definition of ATMP-HE in Pharmaceutical Law applies the broader term “individually prescribed.” Due to the need for Polish authorities to apply a pro-EU interpretation of the Pharmaceutical Law that is consistent with Article 28 of Regulation 1394/2007/EC, the requirement for the product to be individually prescribed should be interpreted in the way that includes the obligation to be custom-made. In Poland the Doctor and Dentist Profession Act regulates the responsibilities of doctors that prescribe ATMP-HE (see **Table 3**). When ATMP-HE are individually prescribed, the doctor is required to verify the individual patient’s needs and therefore this constitutes a custom application of the product.

This table compares the specific provisions of EU Regulation 1394/2007/EC Article 28 versus Polish ATMP-HE regulations under the Polish Pharmaceutical Law.

### 3.2 Principles of using ATMP-HE under Polish Law: perspective of manufacturers

Products to be administered under ATMP-HE regulations in Poland must be manufactured according to specific quality standards. The relevant language of Article 28 of EU Regulation 1394/2007/EC reads as follows: “*Manufacturing of these products shall be authorized by the competent authority of the Member State. Member States shall ensure that national traceability and pharmacovigilance requirements as well as the specific quality standards referred to in this paragraph are equivalent to those provided for at Community level in respect of advanced therapy medicinal products for which authorization is required pursuant to Regulation (EC) No 726/2004/EC of the European Parliament and of the Council of 31 March 2004 laying down Community procedures for the authorization and supervision of medicinal products for human and veterinary use and establishing a European Medicines Agency*” (2, 25). These requirements are adopted into article 38a., 38aa, and 38ab of Polish Pharmaceutical Law (26, 27).

**Table 2** describes the manufacturer responsibilities specified by the Polish Pharmaceutical Law (26, 27). The first step in the chain of quality control is that the manufacturer in Poland must obtain consent for the manufacture of ATMP-HE. Under Polish Pharmaceutical Law, the Main Pharmaceutical Inspector is the authority that is competent to issue consent, refuse to grant it, declare its expiry, or withdraw and change this consent by way of a decision. The manufacturer submits an application to the Main Pharmaceutical Inspector that includes, among others: entrepreneur’s data, list of ATMP-HE, specification of the type and indications of the ATMP-HE, specification of the place of manufacturing in accordance with GMP, scope of activity at the site of manufacture, data of the competent person including specific information about her qualifications, and proof of payment of the fee. If necessary, the application should also include a copy of the manufacturer’s permit for tissue and cell banking.

**Table 2:** ATMP-HE under Polish Law: perspective of manufacturers.

Responsibility	Description
Scope of activity	Manufacturing of ATMP-HE only to the extent covered by the consent.
Indications for use	Application for ATMP-HE was modified by the Polish Minister of Health on February 5, 2019 to oblige the manufacturer to list all possible indications for use of the product, as well as the methods of administration (e.g. intravenous drip, intrathecal injection, etc.) (31).
Notification obligation	Manufacturer must notify the Main Pharmaceutical Inspector in writing, at least 30 days in advance, of any intended change regarding the conditions for manufacturing an ATMP-HE product, and immediately notify the Competent Person.
Obligation to enable inspections	Enabling inspectors to carry out inspections by providing premises, the documentation, and other data regarding the production of ATMP-HE, as well as enabling samples of these products to be taken for quality tests.
Manufacturing entity employs Competent Person	The requirement for the manufacturer to employ a Competent Person was introduced during amendment of the Pharmaceutical Law in 2018 (27). The qualifications of the Competent Person include education at the university level and experience with the production of advanced therapy medicinal products. There are no additional requirements for further education in specific pharmaceutical subjects, because the ATMP-HE products are administered to the patient under the doctor’s liability. The Competent Person is also required to speak Polish to the extent necessary to perform their duties.
Enabling the Competent Person to perform duties	Enabling the Competent Person to perform duties, including making independent decisions within the framework of the powers arising from the regulations.
Application of GMP requirements	Based on the Pharmaceutical Law, the Polish Ministry of Health has stated that “*hospital exceptions must be manufactured in accordance with quality standards equivalent to those for the production of advanced therapy medicinal products with a marketing authorization”* (26).
Public registry^a^	Manufacturers of ATMP-HE are listed on a Polish government website. The Main Pharmaceutical Inspector does not publicize the specific ATMP-HE products and their indications for use.
Reporting Adverse Events	Article 36d of the Pharmaceutical Law requires reporting of every Adverse Event from a medicinal product, and there is no exclusion for ATMP-HE products.
Storing batch release records	Storage of batch release records is required for a period of not less than 30 years from the date of release for use of a given batch of an ATMP-HE product. This requirement is also consistent with the requirements of Art. 34 of the Transplantation Act (32). A long period of time is required because any negative effect of ATMP-HE may not become apparent until many years after its administration.

a- The public list of ATMP-HE manufacturers in Poland is on the web page: Registers of manufacturers, importers and distributors https://www.gov.pl/web/gif/rejestry-wytworcow-importerow-i-dystrybutorow.

This table lists the responsibilities of manufacturers of ATMP-HE in Poland that are specified by the Polish Pharmaceutical Law after the amendment of 2018.

Second, prior to granting or denying consent for manufacture of ATMP-HE, an inspection of the manufacturing facility is conducted by manufacturing inspectors of the Main Pharmaceutical Inspectorate. The purpose of the inspection is to ensure that the entity applying for consent meets the requirements of Good Manufacturing Practice (33). The inspector will determine that: the entity has appropriate premises, technical equipment, and control necessary for the production, control, and storage of ATMP-HE, and that it employs a Competent Person. The qualifications of the Competent Person are listed in **Table 2**. Once these steps have been completed and the consent is granted, the consent is issued for an indefinite period. Follow up inspections are held at least once every two years. Additionally, it is possible to carry out *ad hoc* inspections. It is important to note that the role of the Main Pharmaceutical Inspectorate is purely to ensure the quality of the product and they play no role in judging the medical applications of the product. *“Main Pharmaceutical Inspector does not supervise the selection of therapy and treatment of patients using advanced therapy medicinal products - hospital exceptions (ATMP-HE). … Therefore, it only supervises the conditions of production of the above-mentioned products by inspecting manufacturing sites”* (34).

Third, Article 38ab of Polish Pharmaceutical Law spells out circumstances under which the consent to manufacture ATMP-HE may be withdrawn by the Main Pharmaceutical Inspectorate. These circumstances fall into the categories of obligatory and optional. It is obligatory to withdraw the consent if the manufacturer does not meet some of the statutory requirements. It is optional to withdraw the consent in cases where the possible negative effect on the patient could be disproportionate to the degree of violation of the law. These mechanisms strengthen effective supervision over the area of ATMP-HE production because they introduce rules for withdrawing ATMP-HE consent.

Fourth, we must discuss how Polish regulations interpret the preparation of an ATMP-HE product on a “non-routine basis”, since this is not defined under the umbrella EU regulations (2, 25). The absence of a definition for “non-routine basis” should be regarded as a deliberate omission by the EU regulator which enables the EU member states to make interpretations in the context of each specific healthcare system. In Poland, the national regulator also has not issued any official guidelines or restrictions for “non-routine basis” (26, 27). Each application for ATMP-HE consent is assessed based on the patient’s specific situation.

### 3.3 Principles of using ATMP-HE under Polish Law: perspective of physicians

The Polish Pharmaceutical Law states that ATMP-HE must be administered “*under the exclusive professional responsibility of a physician in order to comply with an individual medical prescription for a given patient*” (26, 27). Unlike the wording of the EU directive which assigns responsibility to a “*medical practitioner*,” in Poland the entire responsibility for administering ATMP-HE rests on the patient’s doctor. The responsibilities of doctors in Poland are spelled out under the Doctor and Dentist Profession Act (29, 30). A doctor in Poland practices a strictly regulated profession, the doctor works within a registered medical entity, and the doctor bears criminal, professional, civil, and sometimes administrative liability. When ATMP-HE are considered as a form of treatment, it is the patient’s doctor that initiates the request to the Bioethics Committee, and once approval is granted the patient’s doctor is responsible for the administration of the therapy. The rules for doctors and the role of the Bioethics Committee are both very important, working in partnership, to regulate the administration of ATMP-HE in Poland.

Since ATMP-HE products have not gone through the full pipeline of clinical trials required for market authorization, their use is subject to the provisions of a therapeutic experiment. The obligations for doctors administering experimental therapy are set forth in Chapter 4 of the Doctor and Dentist Profession Act as follows: “*A therapeutic experiment is the introduction by a doctor of new or only partially tested diagnostic, curative or preventive methods for the purpose of obtaining a direct health benefit for the treated person. It may be conducted if the medical methods used so far are ineffective or not sufficiently effective*” (29, 30). It must be emphasized that the law provides for many obligations related to the commencement of a therapeutic experiment by a doctor, for instance: appropriate purpose of the experiment; appropriate qualifications and proper specialization of the doctor conducting experiment; obtaining informed consent from the patient; and obtaining positive opinion on the therapeutic experiment from a proper Bioethics Committee (35–37).

**Table 3** outlines the specific Polish ATMP-HE regulations provided by the Doctor and Dentist Profession Act, in particular regarding the Bioethics Committee (29, 30). The main principle that should be followed when determining whether a therapeutic experiment is permissible in each case is expressed in Article 22 of the Doctor and Dentist Profession Act. Therapeutic experiments may be carried out if the expected therapeutic benefit may be significant, and the risks associated with achieving this benefit are justified in the light of the current state of knowledge and consistent with the ethical principles of the medical profession. It is worth noting that the current state of medical knowledge is constantly evolving. The amendment of the Doctor and Dentist Act of 2020 created a mechanism to appeal the decision of the Bioethics Committee (30). This policy is consistent with Art. 42 and 42a of the Polish Code of Medical Ethics (38). Art. 28 of the Doctor and Dentist Profession Act states that the data collected may be published for scientific purposes without the consent of the participant, provided the data are anonymized, but there is no legal requirement to publish the outcome data. Retrospective studies of outcomes have been published for ATMP-HE patients in Poland that were treated with MSC for the indications cerebral palsy, spina bifida, and amyotrophic lateral sclerosis (39–41).

**Table 3:** ATMP-HE under Polish Law: perspective of physicians.

Regulation	Description
Qualifications of treating doctor	The doctor should have specialization in a field of medicine that is particularly useful due to the nature or course of the experiment, as well as appropriately high professional and research qualifications.
Requirements for members of the Bioethics Committee	Bioethics Committee members should have high moral authority, high specialist qualifications, and significant experience in matters related to medical experiments. They are obliged to submit declarations of confidentiality and impartiality. A person is excluded from being a commission member if: they have been convicted by a final court judgment of an intentional crime prosecuted by public indictment, or an intentional fiscal crime or legally sentenced to deprivation of the right to practice a profession or a penalty of suspension of the right to practice a profession.
Appointing members of the Bioethics Committee	The Act precisely defines the Institutions authorized to appoint members. Appointments may be made by the district medical council, the rector of a university providing education in the field of medical or health sciences, the director of the State Medical Institute of the Ministry of Internal Affairs and Administration, or the director of an institute of the Polish Academy of Sciences belonging to the Faculty of Medical Sciences
Key elements of a motion before the Bioethics Committee	• designation of the person, persons, entity, or entities intending to conduct a therapeutic experiment; • title of the therapeutic experiment and justification as to its purposefulness and feasibility; • name, surname, address, and professional and scientific qualifications of the person who is to direct the therapeutic experiment; • information about the civil liability insurance of participants; • data on expected therapeutic and possibly cognitive benefits and possibly other expected benefits for participants. • a detailed description of the therapeutic experiment, including the expected number of participants, place, and time of performing the therapeutic experiment, planned procedures, conditions for including and excluding a participant from the medical experiment, reasons for discontinuing the medical experiment and planned use of the results of the therapeutic experiment; • documents confirming the professional and scientific experience of the person who is to lead the therapeutic experiment; • a template of information intended for the participant; • templates of declarations: on the participant’s acceptance of the terms and conditions of civil liability insurance, consent to the processing of participant’s data, and consent of the head of the entity where the medical experiment is planned to be carried out.
Rules for issuing a resolution	The number of Bioethics Committee members may vary. The Bioethics Committee adopts a resolution by a simple majority of votes. The votes are cast by secret ballot and only votes for a positive or negative opinion may be cast. In the event of a tied vote, the chairperson’s vote is decisive. The Bioethics Committee must express its opinion within three months from the date of receipt of complete documentation of the medical experiment.
Rules of appeal proceedings	Since the amendment of July 2020, it is possible to appeal against the resolution of the Bioethics Committee (30). An appeal may be submitted by: the applicant, the head of the hospital where the medical experiment is to be carried out, the bioethics committee responsible for a center that is to participate in a multi-center medical experiment. The appeal procedure is relatively slow: The initial appeal must be submitted within 14 days, if the Bioethics Committee rejects the change, it must send it within 30 days to the Bioethics Appeals Committee, which has 3 months to issue a ruling. The duration of the appeal procedure may be injurious to patients and it remains to be seen if this recent modification is revised again.
Data Collection and Publication	The Doctor and Dentist Profession Act states in Art. 22 that the state of current knowledge is one of the key conditions for conducting a therapeutic experiment. Further, Art. 28 states that the data collected may be published for scientific purposes without the consent of the participant, provided the data are anonymized. However, there is no legal requirement to publish the data.
Sanctions	Violating the regulations regarding conducting therapeutic experiments, in particular regarding conducting them without the required consent of the participant, without the approval of the Bioethics Committee, or contrary to the conditions specified in the application, is subject to criminal sanctions specified in Art. 58 of the Doctor and Dentist Profession Act.

This table lists the responsibilities of doctors prescribing ATMP-HE in Poland that are specified by the Polish regulations regarding therapeutic experiments under the Doctors and Dentists Profession Act.

### 3.4 Principles of using ATMP-HE under Polish Law: perspective of patients

**Table 4** sets out the features required when a patient gives Informed Consent for ATMP-HE therapy. The provisions of Informed Consent for a medical procedure are part of the Doctor and Dentist Profession Act (29, 30). The consent for an experimental procedure contains more provisions than a standard medical consent for a procedure that has market approval. The regulations for ATMP-HE also include safety rules which require that the experiment should be stopped if there is a threat to the participant’s health that exceeds the expected benefits. An update to the original Informed Consent requirements made by Amendment of the Doctor and Dentist Profession Act in 2020 has strengthened the provisions for patient protection (30): Experiments should not be approved if there is evidence from global reports, such as from clinical trials or compassionate use programs conducted elsewhere, that they are not likely to be successful. The participant or the representative acting on their behalf may withdraw consent at any stage of the experiment. Also, participation in the experiment must not delay or deprive the patient from receiving preventive, diagnostic, or therapeutic procedures that are medically necessary.

**Table 4:** ATMP-HE under Polish Law: perspective of patients.

Category	Description
Medical procedures	The plan of the therapeutic experiment must explain its purpose, the scope and duration of the procedures to be used, as well as the potential benefits and risks associated with the experiment.
Rules of financing	An entity conducting an ATMP-HE cannot charge the participant, the participant’s legal representative, or any person who may be directly affected by the effects of the experiment. This rule was established on March 9, 2023 by an amendment to the Act which regulates clinical trials, which takes effect via the Doctor and Dentist Profession Act (42).
Personal information access	These rules govern access to information about the participant that is obtained during the experiment and the analysis of the results.
Compensation rules	These rules spell out the conditions under which the civil liability insurance will pay for damages.
Subsequent medical procedures	After the participant has completed the therapeutic experiment, if it turns out that the experiment brought benefits, these rules cover access to further experimental treatment.
Patients’ rights	The consent should inform the patient of rights and protections that are guaranteed by law, including the right to not give consent and to withdraw consent at any time, without giving a reason and without negative legal consequences.

This table lists the rights of patients receiving ATMP-HE in Poland that are specified by the Polish regulations regarding informed consent under the Doctors and Dentists Profession Act.

Financial restrictions have also been added to the Doctor and Dentist Profession Act which are intended to protect patients receiving experimental therapy in Poland. One such financial rule is that the entity conducting the ATMP-HE must offer the participants civil liability insurance. This was introduced July 16, 2020 by amendment to Article 23c, section 1, of the Doctor and Dentist Profession Act. *“A medical experiment may be conducted after the entity conducting the medical experiment has concluded a civil liability insurance contract for the participant and any other person who may be directly affected by the effects of the experiment”* (30). This provision became effective January 1, 2021 (with exceptions for therapeutic experiments which the Bioethics Committee had begun to consider or had issued an opinion on before that date). An exception to this insurance obligation occurs if the case is urgent and there is a direct threat to the life of the experiment participant.

Another financial restriction was introduced on 9 March 2023 by an amendment to the Act which regulates clinical trials (42). This indirectly alters the enforcement of the Doctor and Dentist Profession Act. This newest restriction prohibits the entity conducting the ATMP-HE experiment from charging fees on participants, the representatives of participants, or people who may be directly affected by the experiment. The justification for this amendment states that this limitation is intended to protect patients against “*abuses by medical entities accompanied by insufficient supervision by bioethics committees*,” because patients themselves are unable to verify the validity of experimental therapy (42). However, it is allowed to obtain funding for ATMP-HE experimental therapies from specialized third parties, such as non-profit foundations, that professionally deal with such activities and therefore have greater knowledge than individual patients (42).

## 4 Discussion

**Table 5** lists several features of ATMP-HE regulations in Poland as compared to three other European countries: Germany, Spain, and the United Kingdom. Together, these four countries are a good illustration of the interplay between unifying concepts versus national differences in the way ATMP-HE are managed in different healthcare systems. Footnotes to **Table 5** cite the breakdown of these parameters in previous studies of ATMP-HE practices in Europe (3–15). It is worth pointing out that many aspects of healthcare in general are not fully harmonized at the EU level. Regarding ATMP-HE specifically, the Article which creates this pathway intentionally uses broad language which allows for differences in the national interpretations of the guidelines (2). Hence, any national regulations that fall within the guidelines cannot be called an improper application, when the original directive is imprecise. It is clear from the columns and footnotes of **Table 5** that ATMP-HE practices in Poland are well within the spectrum of regulatory variations seen among EU countries. For example, if Poland is included, then half of the EU countries surveyed (5 of 10) do not have a guidance to interpret the EU requirement that ATMP-HE must be manufactured under “non-routine” and/or “custom-made” conditions (8). Another example is that that the language of EU Regulation 1394/2007/EC does not restrict the number of patients that may participate in a single ATMP-HE program. This is echoed at the national level, where almost all surveyed EU countries (9 of 10) allow an “unlimited” number of patients to be treated under an ATMP-HE program (8). Thus, in this regard Poland follows the rule and is not an exception. The one aspect in which Poland may be an outlier is the types of payments that are allowed to reimburse the manufacturers of ATMP-HE products. Some countries allow the cost of ATMP-HE to be covered by their national healthcare system, either directly or indirectly through a non-profit connected to the government, whereas Poland requires payment from outside their healthcare system (9).

**Table 5:** How ATMP-HE in Poland compare to three other European countries.

ATMP-HE provision	Poland	Germany	Spain	United Kingdom
Consent to ATMP-HE manufacturer	Chief Pharmaceutical Inspectorate (CPI).	Paul Ehrlich Institute (PEI).	Spanish Agency of Medicines and Medical Devices (AEMPS).	Medicines and Healthcare products Regulatory Agency (MHRA).
Consent to ATMP-HE patient treatment	Doctors and Dentists Profession Act (29, 30).	German Medicinal Products Act, administered by PEI (43, 44).	Each autonomous region in Spain regulates the doctors in their local hospitals (13).	Two exemption schemes coexist, ‘Specials’ and HE. Prescription of ATMP-HE is subject to guidance from the General Medical Council (15, 45, 46).
ATMP-HE “custom-made”^a^	Required, according to art. 2 point 33 b of Pharmaceutical Law. No official guidance (26, 27).	Required. No official guidance (6).	Required. No official guidance (5).	Required. Official Guidance (47).
ATMP-HE made to GMP standards^b^	Yes, according to art. 38aa.1 point 5 of Pharmaceutical Law (26, 27).	Yes (6, 33).	Yes (5, 33).	Yes (48).
ATMP-HE only in Hospitals?^c^	Yes, according to art. 2 point 33b of Pharmaceutical Law (26–28).	No. ATMP-HE can be administered in the outpatient setting in Germany (6).	Yes. Only in the same hospital that manufactured the ATMP-HE product (4, 5).	Yes (15, 46).
Duration ATMP-HE license^d^	Until given up, according to art. 38a.12 of Pharmaceutical Law (26, 27).	Varies, may be renewed (6).	3 years, renew 5 years (5).	Until given up, subject to annual monitoring (46).
Maximum # ATMP-HE patients^e^	No preset limit (26, 27).	No preset limit (6).	No preset limit (15).	No preset limit (15, 46).
Report ATMP-HE Adverse Events^f^	Yes (26, 27).	Yes (6).	Yes (13).	Yes (46).
Payment for ATMP-HE treatment	Costs must be paid by a non-government, non-profit foundation (42).	Costs may be covered by national health insurance (6, 9).	Costs may be covered by national health insurance (4, 9).	Currently costs are covered by hospitals and their subsidiaries, but under proposed new regulations costs may be shared with manufacturers (15).

• a- Regarding the requirement for a “custom made” ATMP product, a previous study found there was no official guidance for this requirement in 4 of 9 EU countries (8).

• b- Regarding whether ATMP-HE must be manufactured to GMP standards, a previous study found this was required in all but 1 of 9 EU countries (8).

• c- Regarding the facilities that may administer ATMP-HE, a previous study found they were restricted to hospitals and public institutions in only 3 of 9 EU countries (8).

• d- Regarding the duration of the HE license, a previous study found that it was limited to a specific number of years in 4 of 9 EU countries (8).

• e- Regarding the number of patients that may receive therapy under an ATMP-HE program, a previous study found this was unlimited in 8 of 9 EU countries (8).

• f- Regarding the reporting of adverse events, a previous study found that this was required in all 7 of 7 EU countries (9).

This table compares ATMP-HE in Poland to three other European countries: Germany, Spain, and the United Kingdom. These countries all have a legal structure where a national pharmaceutical agency grants consent to manufacture ATMP-HE, but the medical practitioner that administers the ATMP-HE answers to an agency that regulates medical professionals. Almost all ATMP-HE practices in Poland are within the mainstream of behaviors seen in previous surveys of seven to nine European countries. The one area where Poland may be an outlier is that the current regulations in Poland forbid the reimbursement of ATMP-HE by either a government-funded institution or by the patient or the patient’s representatives.

It is very important to note that there have been several significant changes to Polish regulations in the past few years, so that current ATMP-HE practices in Poland differ dramatically from cumulative surveys of previous cases (14). In 2018, the Pharmaceutical Law was modified to clarify that ATMP-HE must be administered within hospital healthcare services, both in the sense that hospitals are required to allow ATMP-HE, and in the sense that outpatient facilities cannot offer ATMP-HE (27, 28). Those 2018 modifications to the Pharmaceutical Law also required manufacturers to employ a Competent Person, and spelled out the qualifications and duties of the Competent Person (27). In 2019, the template used when requesting Bioethics Committee approval of ATMP-HE began requiring a description of all indications for use of the product as well as the method of administration (31). In 2020, amendments to the Doctor and Dentist Profession Act created new functions for the Bioethics Committee: the amendment tasked the committee to consider the evidence from previous compassionate use programs when deciding if a treatment is likely to be beneficial, but this amendment also created pathways to appeal decisions of the Bioethics Committee (30). Effective at the beginning of 2021, an amendment to the Doctors and Dentists Profession Act required that participants in ATMP-HE programs must be covered by liability insurance (30). In 2023, further amendments to the section of the Doctors and Dentists Profession Act covering experimental therapies have prohibited ATMP-HE from charging fees on participants (42).

The EU funded a 2018 survey of public perceptions on ATMP therapies under a research initiative called RESTORE (49). This survey of the European public was conducted in 28 countries that were representative of 85.3% of the EU population. Respondents ranked “Healthcare” as the most important topic facing society, ahead of seven other issues including data protection, green energy sources, and migration. Regarding ATMP, the top three findings of the survey were (1): 83% of the surveyed EU citizens support more public funding of technologies in the field of ATMPs (2); 74% of respondents are in support of cross-border ATMP healthcare for patients with rare diseases; and (3) 61% support the reimbursement of very expensive ATMPs within the European health care system despite the current lack of long-term efficacy data (49). Drilling deeper into country-by-country responses to this 2018 survey, citizens in Poland were outliers on some of the issues. Polish people were most likely (50%) to be aware of commercial clinics offering unproven ATMP therapies (49). Polish respondents gave the highest approval (80%) for taxpayer funding of cross-border ATMP healthcare (49). In the published outcomes of ATMP-HE in Poland, patient responses were statistically significant on some metrics and the patients and their families reported satisfaction with their improved quality of life (39–41). Despite the documented enthusiasm of the Polish public for access to ATMP therapies, market uptake of approved ATMP therapies will require that the products be affordable. In the past, many ATMP which received early approvals in EU countries were subsequently withdrawn due to inadequate reimbursements (50, 51).

Affordability of healthcare is a serious concern in Poland. Within the EU, Poland has one of the lowest levels of annual spending per capita on healthcare (49). In 2018 the per capita spending on healthcare was 830 Euro in Poland, whereas for the other countries in **Table 5** the spending was 4627 Euro in Germany, 3646 Euro in UK, and 2310 Euro in Spain (49). Not only do these countries spend between 2.8 and 5.6 times more on healthcare than Poland, but we see from **Table 5** that they also have regulatory frameworks that allow ATMP-HE to be directly or indirectly reimbursed by the government. Surveys of ATMP-HE access have found that only a handful of ATMP-HE have ever been registered in most EU countries, but some ATMP-HE programs have been used to treat hundreds and possibly thousands of patients (7, 9, 51). Until recently in Poland, thousands of patients paid out of pocket to access ATMP-HE at hospitals and clinics. Many of these patients traveled from other countries to take advantage of the ATMP-HE programs in Poland. One manufacturer has compiled a registry of over 3450 patients treated, from 35 countries, primarily with MSC, in both clinical trials and ATMP-HE programs (52). But, with the overhaul of Polish regulations during the years 2018 – 2023, this direct-to-consumer access to ATMP-HE has stopped. At present, manufacturers that wish to provide ATMP-HE in Poland are reorganizing to run these programs exclusively in hospitals and funded solely by third party payers (27, 42).

The economic impact of ATMP-HE regulations in Poland is neutral from the perspective of public expenditures on the healthcare system. Whether the ATMP-HE are financed directly by patients or through non-profit foundations, in either case the costs do not burden the budget of the National Health Fund, which is underfinanced. In addition, Polish regulations specify that ATMP-HE can only be used if existing registered therapies cannot meet a patient’s needs, so there is no competition with approved therapies. At the same time, the effective ATMP-HE programs are an economic benefit to society as a whole, since any improvement in the quality of life for patients with disabilities lessens their burden on their families and society. The decision to prevent patients from spending their own money on these treatments has limited their autonomy to pay for a health service which is not paid for by public healthcare programs. This decision may be in legal conflict with provisions that protect individual rights. It could be argued that the existing provisions of the Doctor and Dentist Profession Act, which require the Bioethics Committees to only approve therapies that are likely to be beneficial, should prevent abuse if implemented correctly. In the meantime, the need to reorganize ATMP-HE programs in Poland around third party payers has limited patient access, because some treatments have been suspended temporarily. For example, Poland had the only ATMP-HE program that provided MSC infusions to children with autism, and this program is currently not recruiting.

The motivation of the original EU Regulation 1394/2007/EC was to provide expanded access to experimental therapies for patients with unmet needs. This can be illustrated with two diagnoses that carry a high cost of disability and which have been treated in Poland with ATMP-HE that employ MSC. One example is the adult orthopedic condition knee osteoarthritis, which has a prevalence of 3.6% among the 448 million people in the EU, or 16 million patients (53, 54). Another example is the pediatric neurologic condition autism, which has a prevalence of 1.4% among the 80 million children under age 18 in the EU, or 1.12 million patients (55, 56). Merely for these two conditions, large numbers of patients across Europe might be eligible for expanded access to MSC therapies via ATMP-HE. These ATMP could be manufactured in a network of regional centers of excellence, as some have proposed (12, 57). Thus, for these diagnoses alone the number of patients with unmet needs vastly exceeds the number of enrollments available in clinical trials or the number of openings that have been available in ATMP-HE programs. Nonetheless, the European Federation of Pharmaceutical Industries and Associations (EFPIA), along with other organizations that represent pharmaceutical companies, has issued a joint position paper calling for modification of EU regulations on ATMP-HE (58). Modifications that improve the quality of care are welcome, but the list of proposed revisions must be considered carefully to avoid limiting patient access unnecessarily. For example, if there is a strict prohibition that no patient may obtain ATMP-HE when a similar clinical trial exists, regardless of the enrollment size of the trial, then many patients will be cut off from legal access to MSC treatment. It remains to be seen if the stakeholders in ATMP-HE, both within Poland and in other EU countries, can find a middle ground between protecting patients from exploitation versus allowing patients with incurable conditions and unmet needs to have access to experimental therapies.

## 5 Conclusions

To date there is no full harmonization of ATMP-HE regulations among EU countries, beyond the overall guidelines provided in the original EU regulation that created ATMP-HE (2–15). We have demonstrated that the Polish implementation of the EU directive is within the mainstream of practices among EU countries regarding manufacturing of products and management of ATMP-HE programs. In addition, between the years 2021 and 2023 Poland has implemented very strong policies for patient protection. The regulations that govern the conduct of therapeutic experiments now require that ATMP-HE participants must have civil liability insurance, and prohibit patients or their representatives from paying to participate in ATMP-HE. It is remarkable that while the public in Poland has among the lowest EU levels of government spending on healthcare, they have among the highest EU levels of public awareness of ATMP and desire to access ATMP.

## Data availability statement

The original contributions presented in the study are included in the article/supplementary material. Further inquiries can be directed to the corresponding authors.

## Author contributions

JP: Conceptualization, Data curation, Formal analysis, Funding acquisition, Writing – original draft. FV: Conceptualization, Data curation, Formal analysis, Visualization, Writing – original draft, Writing – review & editing.
